# Effect of Pre-Wetted Zeolite Sands on the Autogenous Shrinkage and Strength of Ultra-High-Performance Concrete

**DOI:** 10.3390/ma13102356

**Published:** 2020-05-20

**Authors:** Guang-Zhu Zhang, Xiao-Yong Wang

**Affiliations:** Department of Architectural Engineering, Kangwon National University, Chuncheon-Si 24341, Korea; zhangks@kangwon.ac.kr

**Keywords:** calcined zeolite sand, ultra-high-performance concrete, pre-wetted, autogenous shrinkage, internal curing

## Abstract

In this study, the carrier effect of zeolite sands in reducing the autogenous shrinkage and optimizing the microstructure of ultra-high-performance concrete (UHPC) is studied. Pre-wetted calcined zeolite sand (CZ), calcined at 500 °C for 30 min, and natural zeolite sand (NZ), with 15 wt.% and 30 wt.% in UHPC, are used to partially replace standard sands. On that basis, a series of experiments are executed on the developed UHPC, including compressive strength, autogenous shrinkage, X-ray diffraction (XRD), and isothermal calorimetry experiments. With the increase of the zeolite sand content, the autogenous shrinkage of UHPC decreases gradually. Moreover, when the added CZ content is 30 wt.% (CZ30 specimen), it is effective in reducing autogenous shrinkage. Meanwhile, at the age of 28 days, the compressive strength of CZ30 is 97% of the control group. In summary, it is possible to effectively reduce the autogenous shrinkage of UHPC containing 30 wt.% CZ, without sacrificing its mechanical properties.

## 1. Introduction

In recent years, due to the increasing demand for concrete, ordinary and high-performance concrete (HPC) may not meet actual engineering needs. Thus, ultra-high-performance concrete (UHPC) is of increasing interest to researchers. Compared with ordinary concrete, UHPC shows a more dense microstructure and excellent compressive strength, because it contains a large amount of cement-based materials, has a low water/binder (w/b) ratio, and uses a large number of superplasticizers [1,2]. In addition, the durability of ultra-high-performance concrete has been significantly improved, so UHPC could be used under more severe conditions. However, it must be considered that, due to the influence of a range of factors—such as a high cementitious material content and low w/b ratio—UHPC experiences autogenous shrinkage at an early age [3]. Compared with ordinary concrete, UHPC is more prone to cracking in the early stage [4]. The influence of early cracking, caused by restrained autogenous shrinkage, even limits the application of UHPC [5,6]. Therefore, solving the problem of the autogenous shrinkage of UHPC is urgently required.

Researchers have studied the following five methods to reduce the occurrence of the autogenous shrinkage of UHPC: (1) the control of the hydration reaction; (2) reduction of the internal restraint; (3) reduction of the surface tension of the pore solution; (4) formation of expansive products; and (5) replenishment of water through internal curing. Of these five methods, internal curing is considered to be the most effective and straightforward method [7]. Superabsorbent polymer (SAP) [8,9] and lightweight aggregates (LWA) [10,11] are considered to be two common internal curing materials. While SAP is very effective in reducing autogenous shrinkage, it also introduces additional pores into the concrete structure due to its water swelling effect, resulting in a reduction in the compressive strength of the concrete structure [12]. It was confirmed by Sun et al. [13] that the incorporation of SAP reduced the compressive strength, and the compressive strength decreased with the addition of SAP. In the study of Farzanian et al. [14], it was also concluded that the addition of SAP reduces the compressive strength of cement slurry in cement pastes with a high density of macrovoids. However, in other studies of concrete mixed with SAP, some researchers have come to the opposite conclusion. Bentz et al. [15] measured compressive strength development in experiments carried out for mortar mixes with w/b = 0.35, with and without SAP (0.4% relative to binder mass). After 28 days of curing, mortar with SAP showed higher compressive strength than the reference mortar; the values were 73 and 61 MPa, respectively. Similarly, Woyciechowski and Kalinowski [16] studied the influence of the dosing method of SAP on the effectiveness of the concrete. It was found that the 28-day compressive strength of concrete with an activated small particle size (150–850 µm) of SAP was higher than that of the control specimen. In summary, the effect of SAP on strength depends on the system being studied. Compared with SAP, LWA can reduce the autogenous shrinkage of concrete, but whether the compressive strength of the concrete structure is reduced depends on the type of LWA material [17]. Wang et al. [18] studied the effects of three different types of pre-wetted LWA (fly ash-clay ceramsite, shale ceramsite, and clay ceramsite) on the compressive strength and shrinkage of concrete and found that LWA will reduce the compressive strength of concrete. Liu et al. [10] also studied the effect of saturated coral aggregate (SCA) with UHPC and found that, although the autogenous shrinkage is reduced, the mechanical properties are also lost. The most widely used materials for LWA are different types of sand. However, the LWA material used in this paper is zeolite sand. Zeolite sand not only can effectively change the mechanical properties of concrete structures, but is also an environmentally friendly material that can be used for gas purification [19,20]. While ordinary natural zeolite sand can absorb a certain amount of water due to its fine pore structure, it has difficulty in completely releasing most of the absorbed water. The crystal structure of zeolite sands can be destroyed by heat treatment to significantly increase their water absorption capacity, while the zeolite sand porosity is changed by particle agglomeration [21]. According to the literature [22], increasing the water absorption of zeolite sand by calcination is very effective. Zhang et al. [23] used calcined zeolite particles with an average size of 0.18 mm as the internal curing agent of high-strength concrete, and it was confirmed that the calcined zeolite increased the internal relative humidity of the concrete and reduced the shrinkage. Zhang et al. [24] also applied pre-wetted calcined zeolite particles in a high-strength engineered cementitious composite, and more than 60% of autogenous and/or drying shrinkage at 28 days was reduced while the strength of the composite remained as high as the reference specimen. Some zeolite powders were also used to mitigate the autogenous shrinkage of concrete. Pezeshkian et al. [25] studied the effect of different percentages of silica fume replaced with natural zeolite powder (25%, 50%, 75%, and 100%) on the autogenous shrinkages and mechanical properties of UHPCs. It showed that with UHPC in which 50% in volume of natural zeolite was used as a substitute for silica fume, the 28- and 90-day compressive strengths were only slightly lower than that of reference specimen. Meanwhile, an increasing number of researchers have found that fine lightweight aggregates can react. Li et al. [26] analyzed the pore solution and found that the expanded shale and clay can reduce the alkalinity, as well as increase the aluminum content, in the pore solution. Suraneni et al. [27] found that finely ground lightweight aggregate is pozzolanic and participates in the hydration. In this paper, we also found that zeolite sand is not inert.

In practical engineering applications, most construction workers are highly interested in maintaining a similar compressive strength while reducing the autogenous shrinkage of concrete. The aim of this paper is to use pre-wetted zeolite sand to replace a portion of the standard sand in UHPC in order to significantly reduce the autogenous shrinkage of UHPC, without a significant loss of strength caused by adding the zeolite sand. The research methods implemented include water absorption, autogenous shrinkage, compressive strength, X-ray diffraction, and isothermal calorimetry tests.

The innovations of this paper are summarized as follows: Firstly, we find the use of 30 wt.% calcined zeolite sand can reduce the autogenous shrinkage of UHPC without reducing its compressive strength. Secondly, we find zeolite sand is not chemically inert, and the dissolution of fine zeolite sand particles changes the alkali ion concentration of the solution, accelerates the early binder hydration, and promotes the setting of the binder. Finally, the calcination can increase the water-absorbing capacity of zeolite sand and the internal curing effect of zeolite sand is enhanced after calcination.

## 2. Materials and Methods

### 2.1. Materials and Sample Preparation

**Figure 1:**
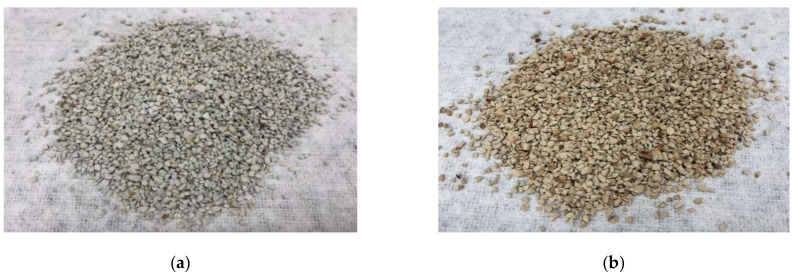
Zeolite sands used to replace standard sand: (**a**) natural zeolite sand; (**b**) calcined zeolite sand.

In this paper, zeolite sands with minimum and maximum particle sizes of 1 mm and 3 mm, respectively, were used as internal curing agents of UHPC. To observe the influence of calcined zeolite sand on UHPC, natural zeolite sand particles were heated for 30 min in a muffle furnace at 500 °C. Figure 1 shows calcined zeolite sand after being calcined by a muffle furnace. The binder materials included Type I ordinary Portland cement (OPC) and silica fume (SF).

The chemical compositions of cement, silica fume, and zeolite sand, measured by X-ray fluorescence (XRF), are shown in Table 1. 

The XRF fused cast bead method (ISO 12677:2003) was used in the sample preparation (XRF-Scientific, Tokyo, Japan). Samples of lithium tetraborate and lithium metaborate (each ~4 g) and a test sample (~0.8 g) were mixed for preparing the beads. The resistance furnace was heated to a fixed temperature of 1025 ± 25 °C [28].

**Figure 2:**
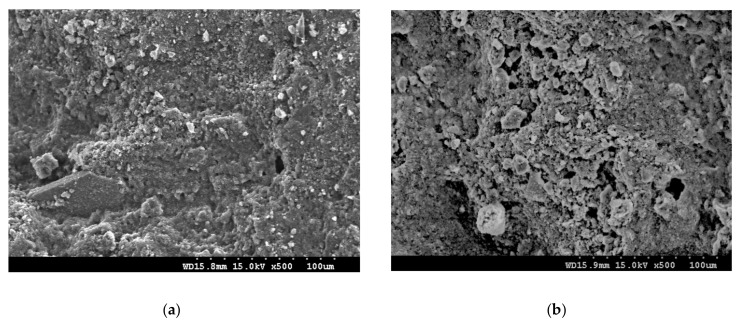
SEM images: (**a**) natural zeolite sand; (**b**) calcined zeolite sand.

The SEM images of the natural zeolite sand and calcined zeolite sand are shown in Figure 2. 

Microscopic observation of the sheet samples was performed using a high-resolution field emission scanning electron microscope (S-4300, Hitachi, Tokyo, Japan) with an acceleration voltage of 1.5 kV and an emission current of 7000 nA. The zeolite sands were broken, and the inner sheet was selected for testing. Prior to microscopic observation, the surface of the samples was coated with platinum for 30 min using a Hitachi E-1010 ion sputterer (Tokyo, Japan) [28]. As shown in Figure 2, compared with natural zeolite sand, calcined zeolite sand has more pores and even a greater porosity distribution. A fine aggregate (standard sand), with a maximum particle size of 3.0 mm, was used.

To achieve the objectives of this study, two sets of tests were conducted. In the first test group, the natural zeolite sand was used as the internal curing agent, and the additional amounts were 15 wt.% and 30 wt.% of standard sand, respectively. The UHPC specimens are marked as NZ15 and NZ30. In the second test group, calcined zeolite sand was used as an internal curing agent, and the additional amounts ware 15 wt.% and 30 wt.% of standard sand, respectively. The UHPC specimens are marked as CZ15 and CZ30. The water absorbed by the zeolite sands, before setting, has no effect on the porosity of the concrete and is therefore not considered to be part of the ‘water/cement ratio’ of a particular concrete mixture [29]. The mixture proportions of the UHPC specimens are shown in Table 2.

This paper focuses on the internal curing effect of zeolite sand on strength and autogenous shrinkage. Since steel fiber is mainly used to improve the ductility of UHPC, and ductility is not the aim of this paper, we did not use fiber.

**Figure 3:**
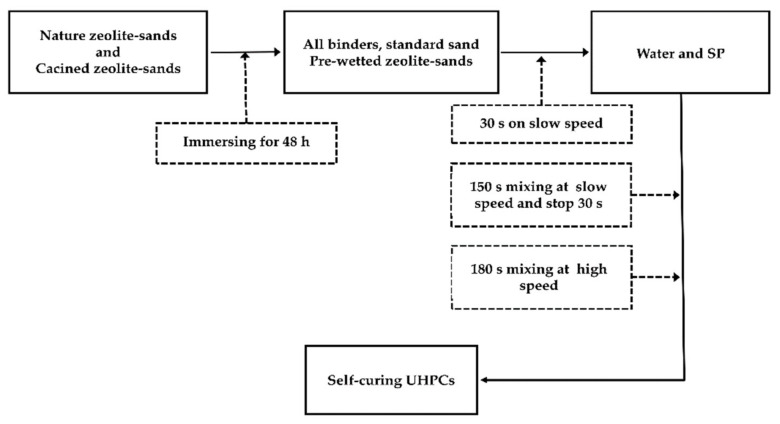
Detailed information on the mixing procedure adopted to produce self-curing UHPCs.

Figure 3 shows detailed information on the mixing procedure, adopted to produce self-curing UHPCs. 

The natural zeolite sand and calcined zeolite sand, soaked in water for 48 h, were taken out. The soaked zeolite sands were unfolded with a paper towel, and a paper towel was placed on the surface of the zeolite sands to dry the surface of the zeolite sands [30]. The above steps were repeated until a color change in the paper towel could not be visually detected. At this point, it was assumed that the zeolite sands had reached a surface drying state [31]. All binders, standard sand, and zeolite sands are placed in a mortar mixer and stirred at a low speed for 30 s. Water, uniformly mixed with SP, was added to the solid material and stirred at a low speed for 150 s. The process was stopped for 30 s, and the powder that was not sufficiently contacted on the paddles and inner wall of the mortar mixer was scraped. The mixture was further stirred at a high speed for 180 s, and then the mortar was poured into a mold for shaping and curing.

### 2.2. Test Methods

#### 2.2.1. Water Absorption

Since the absorption of water by the zeolite sand specimens occurs over time, to determine the absorption of water changing with time, the volumetric flask test was used to determine the saturation of the zeolite sand specimens for 24 h. The specimens were placed in an oven at 105 °C for 24 h and cooled for another 24 h. The measuring method of water absorption was the same as that used in [32]. Of the sample, 100 g was put into a 250 mL volumetric flask. Deionized water was added to about 80% of the volumetric flask, which was then stirred manually for 2–3 min to remove bubbles between the zeolite sands. After stirring, and after the zeolite sand particles were submerged, the horizontal position of the liquid in the volumetric flask was observed, and extra water was added to make the water level in the volumetric flask reach its pre-marked standard horizontal position. As the deionized water was continuously absorbed by the zeolite sand particles, the level of the liquid decreased. Within the specified time intervals (approximately 10 min, 20 min, 30 min, 1 h, 3 h, 4 h, 5 h, 6 h, 24 h, 36 h, and 48 h), additional water was added to the flask to ensure that the liquid level reached the standard calibration. The total quality and actual measurement time were recorded. It should be noted that the flask needed to be stirred for about 30 s to remove the bubbles between the zeolite sand particles before each addition of water.

#### 2.2.2. Autogenous Shrinkage

A corrugated tube method, based on ASTM C1698–09 [33], was used to measure the linear autogenous shrinkage of cement mortars. A corrugated tube with concrete was placed on the steel support, and one end of the corrugated tube was absorbed by a magnet. The other end was in contact with a linear variable differential transducer (LVDT) to measure the deformation throughout the experiment. The LVDT collected data through computation every 10 min. The whole setup was maintained in a constant temperature humidity chamber at 20 °C, until the test age of 7 days. It should be noted that in order to avoid a large quantity of entrapped air in the test specimens, mixtures were slowly poured into the corrugated mold with the vibrating table turned on.

#### 2.2.3. Compressive Strength Test

The compressive strength of the designed UHPC was tested (Shin Gang, Seoul, Korea), based on the ASTM C39 [34]. The cement mortars were manufactured as a standard specimen, with a size of 50 mm × 50 mm × 50 mm, and the compressive strengths at 3, 7, and 28 d were measured. All specimens were sealed and cured at a temperature of 20 °C.

#### 2.2.4. X-Ray Diffraction

X-ray powder diffraction (XRD) analysis was conducted on the raw materials (natural zeolite sand and calcined zeolite sand) and the samples (after 28 days of curing) after the hydration stoppage treatment. The solvent exchange method was used for hydration stoppage of the hydrated samples [35]. We ground the 5 g of hydrated samples into a powder. The powder was then immersed in 150 mL of isopropanol (CP grade) for 15 min. The suspension was vacuum filtered, rinsed once with isopropanol, and rinsed twice with ether (CP grade). The filtered solids were dried in a drying oven at 40 °C for 8 min. Finally, the dried powder was stored in a vacuum dryer. Testing of samples should be performed as soon as possible, preferably within two days. The raw materials and samples were ground until the sample surface was flat and smooth. The sample surface area and thickness was large enough to avoid beam overflow and sample transparency aberrations [36]. XRD was carried out using a Panalytical X’pert-pro MPD diffractometer (Panalytical, Almelo, The Netherlands), with CuKα radiation (λ = 1.5404 Å) [37]. The measurements were conducted in the 2θ range of 5°–70°, with a 2θ scan rate of 0.1°/min.

#### 2.2.5. Isothermal Calorimetry

In previous studies, most of the specimens for isothermal calorimetry were pastes, without a fine aggregate. These studies ignored the effect of fine aggregates on the heat of hydration. Conversely, in this study, to clarify the effect of fine zeolite aggregates on the heat of hydration, a mortar specimen with a fine zeolite aggregate was used for isothermal calorimetry. The heat release rate and accumulated hydration heat of the adhesives were measured using a TAM-Air (TA Instruments, New Castle, DE, USA) for 72 h at 20 °C. Due to the low water binders (w/b = 0.2), all mortars were mixed by a mortar mixer and transferred to glass bottles [38].

## 3. Results and Discussions

### 3.1. Water Absorption

**Figure 4:**
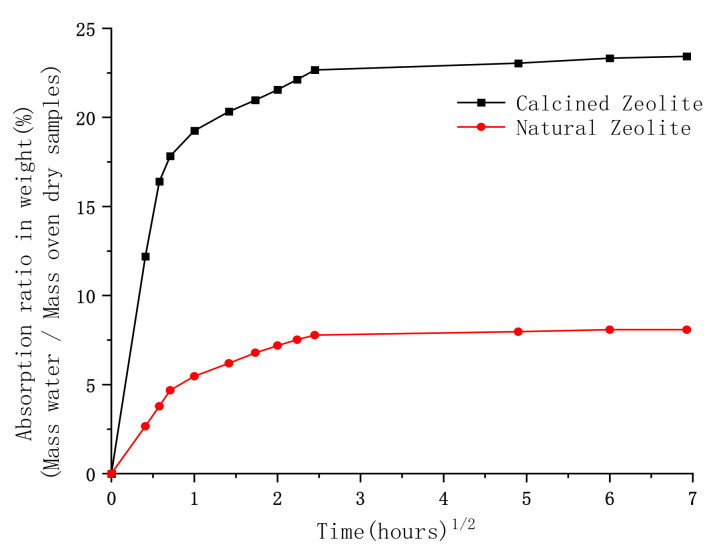
Square root of time vs. water absorption of zeolite sands, before and after calcining.

Figure 4 shows the water absorption after the natural zeolite sand and the calcined zeolite sand had been dried for 48 h. Both zeolite sands absorbed most of the water within the first 6 h. 

The water absorption of the natural zeolite sand reached 7.78% in the first 6 h, while the calcined zeolite sand reached 22.67%. The total water absorption increased with time, and the final water absorption of the natural zeolite sand reached 8.08%, while the calcined zeolite sand reached 23.43%. The water absorption of the calcined zeolite sand was higher than that of the natural zeolite sand. This is due to the initial physical bonding water present in the natural zeolite sand being evaporated during heating, resulting in more pores becoming empty. Similar results were obtained in [39]. The experimental results are in good agreement with the SEM images, shown in Figure 2.

### 3.2. Autogenous Shrinkage

**Figure 5:**
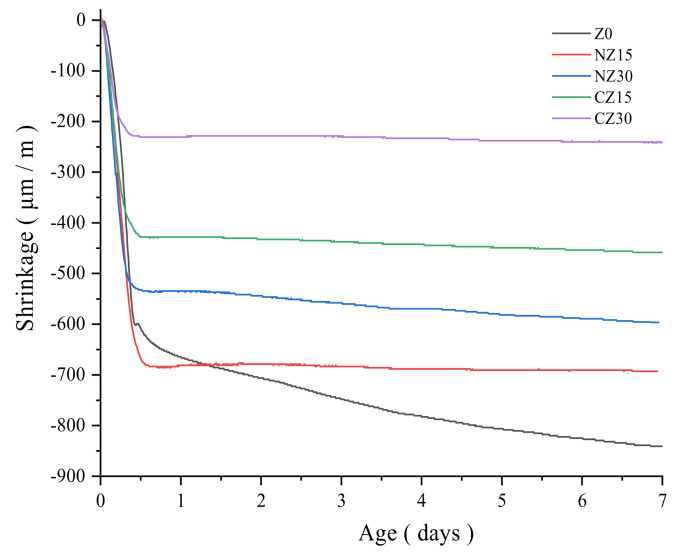
Development of the autogenous shrinkage of all mixtures for 7 days.

All the mortars are placed in a corrugated tube, and the autogenous shrinkage of the mortars, during the 7-day period, was measured using the deformation measurement setup. The test data are shown in Figure 5. 

The control group is labeled as Z0, and the test pieces with the calcined zeolite sand, containing 15 wt.% and 30 wt.%, are labeled as CZ15 and CZ30, respectively. The test pieces with natural zeolite sand, containing 15 wt.% and 30 wt.%, are labeled as NZ15 and NZ30, respectively. It is observed in Figure 5 that the overall trend is that the shrinkage of the specimens decreases with the increased number of zeolite sand particles. Because zeolite sand absorbs additional water as a self-curing agent, parts of the self-curing agents are released with the binder hydration, and the released internal curing water fills the air space formed inside the UHPC due to chemical shrinkage, reducing the occurrence of autogenous shrinkage strain [40].

First, the shrinkage of Z0 increases rapidly within the first 12 h and slowly increases after 12 h. However, specimens containing calcined or natural zeolite sand remain unchanged after the inflection point. This is because the addition of zeolite sand is equivalent to adding a reservoir to the cement and, with the binder hydration, the internal curing water of zeolite sand is released and continues to participate in the hydration reaction, thus slowing down the occurrence of autogenous shrinkage. Similar results have been confirmed in previous studies [39,41].

Second, calcined zeolite sand is more effective in reducing the autogenous shrinkage of the specimens. The shrinkage of the specimens containing calcined zeolite sand (CZ15 = −458.293 μm/m, CZ30 = −239.947 μm/m) is lower than that of specimens containing natural zeolite sand (NZ15 = −692.952 μm/m, NZ30 = −595.542 μm/m). This means that the modification of zeolites is more helpful in improving the storage capacity of pores. While the specimens contain the same amount of zeolite sand, the calcined zeolite sand absorbed more internal curing water than the natural zeolite sand. The specimens containing the calcined zeolite sand released more internal curing water, and the released internal curing water increased the relative humidity inside the UHPC. Consequently, the shrinkage of the specimens with calcined zeolite sand is smaller than that of the specimens with natural zeolite sand.

### 3.3. X-Ray Diffraction (XRD)

**Figure 6:**
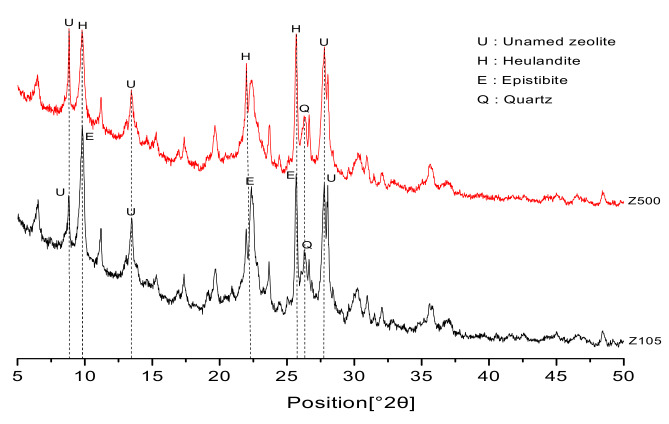
XRD patterns of natural zeolite sand and calcined zeolite sand.

XRD measurements were conducted on both the natural zeolite sand and calcined zeolite sand, and the experimental results are shown in Figure 6. 

Calcined zeolite sand belongs to the heulandite series, which includes a kind of thermally stable heulandite zeolite (CaO·Al_2_O_3_·7SiO_2_·5H_2_O), synthesized by CaO·Al_2_O_3_·7SiO_2_ in the temperature range of 250–360 °C. The calcined zeolite sand belongs to the epistilbite series (CaO·Al_2_O_3_·5.5SiO_2_·5H_2_O), synthesized by the CaO·Al_2_O_3_·5.5SiO_2_ composite [42]. Due to the change of temperature during the calcination process, the crystal shapes of the zeolites are changed, but there is no change in the chemical compositions. Previous studies have shown that calcining natural zeolite will lead to a collapse of crystal structures [43]. There is also an unnamed zeolite phase and mineral impurities, such as quartz.

**Figure 7:**
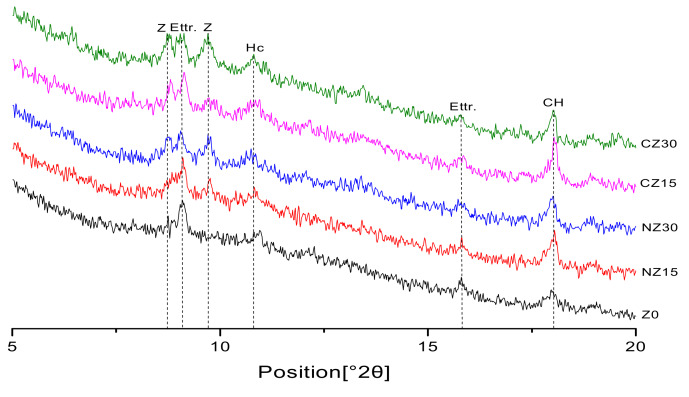
XRD patterns of UHPC casting for 28 days.

The XRD patterns of mortars at the curing age of 28 days are presented in Figure 7. 

Since the diffraction peak of the sand would cause interference, a 2θ degree between 5 and 20° is selected. Compared with the control group, no new hydration products are generated, because no new peaks appeared. This is mainly because zeolite sands, as lightweight aggregates added to concretes, do not participate in a binder hydration reaction.

According to the peak of calcium hydroxide in the Figure 7, compared with the control group, Z0, the peak of calcium hydroxide increased significantly with the addition of zeolite sand. 

This is because the specimens containing zeolite sands contain more internal curing water [44]. With the occurrence of the cement hydration reaction, the water in the reservoir is released to further promote cement hydration, and more hydration products are obtained. Since zeolite sands are placed into the system as lightweight aggregates, the zeolite sands themselves do not participate in the early cement hydration reactions. The Ca(OH)_2_ peak of UHPC, containing calcined zeolite sand, is higher than that containing natural zeolite sand, mixed with the same content. This is because the calcined zeolite sand can absorb more internal curing water, which is later released to further participate in a hydration reaction. More hydration products are produced, leading to an increase in the peaks of the hydration products. The difference between the corresponding Ca(OH)_2_ peaks in CZ15/30 and those in NZ15/30 are not obvious. XRD qualitative analysis method can determine the type and relative quantity of hydration productions, but it cannot determine the specific amount of hydration products. Therefore, the XRD qualitative analysis is not the best method of analyzing the degree of hydration. TGA can be used as a quantitative analysis method to measure the content of chemically bound water and calcium hydroxide. Compared with XRD qualitative analysis, TGA is a much better way to determine the degree of hydration, and a TGA experimental study will be conducted in the future.

### 3.4. Isothermal Calorimetry

All mortars were mixed by a mortar mixer and transferred to a TAM-Air. The heat release rate and accumulated hydration heat of the adhesives were measured using a TAM-Air for 72 h at 20 °C. According to the hydration heat release curve of mortars, the cement hydration could be divided into five periods: (1) initial (pre-induction) period; (2) induction period; (3) acceleration (post-induction) period; (4) deceleration period; and (5) diffusion period [45]. Because the mortar mixing takes place outside the glass bottles, it is difficult to fully capture the peak of the first period. In this study, Z0 represents a control group; CZ15 and CZ30 represent UHPCs doped with 15 wt.% and 30 wt.% of calcined zeolite sand, respectively; and NZ15 and NZ30 represent UHPC doped with 15 wt.% and 30 wt.% of natural zeolite sand, respectively.

**Figure 8:**
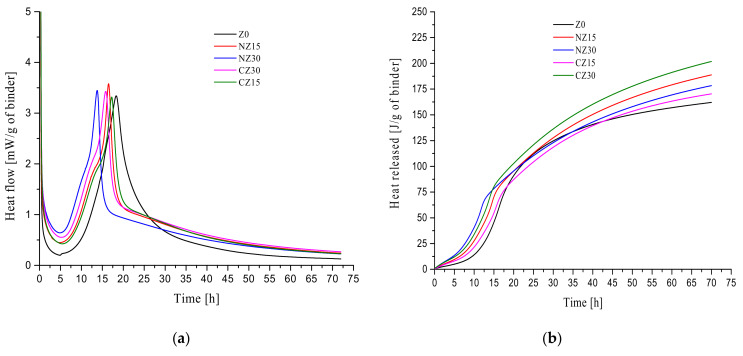
Isothermal calorimetry curves of UHPCs for 72 h: (**a**) heat flow curves; (**b**) cumulative heat curves.

As shown in Figure 8a, in the initial period, compared with the control group, Z0, the heat release rate of self-curing UHPC increased because of the addition of zeolite sand. 

For the UHPC containing calcined zeolite sand, as the content of calcined zeolite sand increased, the heat release rate of mortars accelerated. The heat release rate of mortars containing 30 wt.% of zeolite sand was the highest in the same group, which may be due to the addition of a large amount of zeolite sand, since soluble zeolite sand particles increase the content of alkali ions [1]. The same phenomenon is observed in mortars containing natural zeolite sand.

In the acceleration period, the time at which heat flow peaks of each hydration heat level appear are in the order of NZ30 > CZ30 > NZ15 > CZ15 > Z0. This is because some small zeolite sand particles are dissolved, and the alkali ion concentration is increased, which accelerates the binder hydration, causing the setting time to increase. During the deceleration period, it could be observed that the slope of the heat release rate curve of CZ15 was the same as that of CZ30 with respect to the hydration reaction. The slope of the heat release rate curve of NZ15 was the same as that of NZ30 with respect to the hydration reaction. Finally, in the diffusion period, the heat release rates of the mortars containing calcined zeolite sand and natural zeolite sand were higher than those of the control group, Z0.

Figure 8b describes the total heat release of the mortars within 72 h. It can be observed that the total heat released from self-curing UHPC is higher than that of the control group, Z0. The heat release of CZ30 is 260.84 J/g, which is the highest among all specimens. For the mortars mixed with natural zeolite sand, the heat release of NZ30 is higher than that of NZ15 at the initial stage of hydration, and the intersection point appears at about 20 h. After the intersection point, the heat release of NZ15 is higher than that of NZ30 at the later stage of hydration. At the age of 3 days, the heat release of NZ15 (188.71 J/g) is greater than that of NZ30 (178.81 J/g), which is caused by the heat release rate of NZ15 in the hardening deceleration stage being higher than that of NZ30.

### 3.5. Compressive Strength

**Figure 9:**
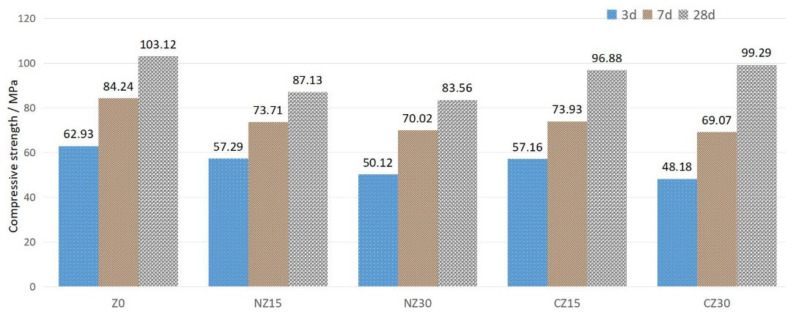
Compressive strength change of UHPCs at 3, 7, and 28 days.

The compressive strength, measured by cube specimens with dimensions of 50 mm × 50 mm × 50 mm, of UHPC at 3, 7, and 28 days, is shown in Figure 9.

At an age of 3 days, compared with the Z0, with the addition of zeolite sand, the compressive strength decreased. This is because zeolite sand is a porous material, and the addition of zeolite sand increases the porosity of concrete. Moreover, with the content of natural zeolite sand increasing from 15 wt.% to 30 wt.%, the compressive strength decreased because of the negative effect of porous zeolite sands. The compressive strengths of the NZ15 and NZ30 specimens are similar to those of CZ15 and CZ30 at early ages.

However, up to the age of 28 days, the compressive strengths of NZ15 (87.13 MPa) and NZ30 (83.56 MPa) are smaller (15.51% and 18.97%) than those of Z0 (103.12 MPa). The negative effect seems to have been compensated for. The compressive strength of CZ30 increases significantly, achieving 99.29 MPa, which is close to the compressive strength of Z0—about 103.12 MPa. The compressive strengths of CZ15 and CZ30 are 6% and 3.7%, which are lower than that of Z0. This may be due to the release of internal curing water in the zeolite sands, which fills the pores in the zeolite sands and improves the compressive strength of UHPC [39].

From 3 to 28 days, the incremental degree of strength for Z0 and CZ30 is different due to the effect of internal curing. The incremental degree of strength equals the ratio of the strength at 28 days to the strength at 3 days, and, regarding the increment degree of strength, Z0 increased by 1.64 (103.12/62.93 MPa) and CZ30 increased by 2.06 (99.29/48.18 MPa). The compressive strength of CZ30 increased more obviously. This is mainly because the addition of zeolite sand increased the porosity of the specimens. However, as the hydration reaction proceeds, the release of the internal curing water of the reservoir promotes the hydration of the cement, which significantly increases the compressive strength of the specimen at a later age. Therefore, the use of calcined zeolite sand as an autogenous curing agent has no obvious impairing effect on the compressive strength of UHPC.

### 3.6. Discussion

UHPC can be divided into two types, namely, precast UPHC and cast-in-place UHPC. For cast-in-place UHPC, autogenous shrinkage is an essential constraint because UHPC has a very low water-to-binder ratio. Construction companies are urgently seeking a solution to mitigate or minimize autogenous shrinkage without impairing the strength. In this study, we find the calcined zeolite sand is an effective internal curing material for UHPC. Compared with the control specimen, the autogenous shrinkage of concrete with 30% calcined zeolite sand is significantly lower while the 28-day compressive strength is similar.

On the other hand, the compressive strength of the UHPC designed in this paper did not reach 150 MPa (the general strength of UHPC). This may due to many factors affecting the strength of UHPC, such as: fibers [46], binder composition [47], particle size distribution, compactness of aggregate, and curing procedure [48]. The addition of fiber can improve the strength and ductility of concrete. The early-age high temperature curing may also contribute to the strength increment of concrete [49]. In addition, calcined-zeolite sand has some weak points; for example, the intrinsic porosity of the calcined zeolite sand may decrease the compactness of UHPC. These weak points should be further studied in future research [11,50].

## 4. Conclusions

Studying the effects of natural zeolite sand and calcined zeolite sand on the properties of UHPC, the following conclusions can be drawn from the present study:

1. Compared with natural zeolite sand, the water absorption of calcined zeolite sand is obviously increased. This is due to the initial physical bonding water present in the natural zeolite sand being evaporated during heating, resulting in more pores becoming empty.
2. The autogenous shrinkage of self-curing UHPC is significantly reduced due to the addition of zeolite sands. Among the resulting outcomes, the effect of adding calcined zeolite sand to UHPC is the most obvious. This is because calcined zeolite sand can absorb more internal curing water before mixing. With the occurrence of the hydration reaction, internal curing water is released to continue to participate in the hydration reaction, thereby reducing autogenous shrinkage.
3. For specimens with zeolite sand additions, XRD analysis showed that there were no new hydration-product peaks, indicating that zeolite sand particles do not participate in binder hydration. The peak of calcium hydroxide increased significantly with the addition of the zeolite sands. This is because the specimens containing zeolite sands contain more internal curing water. With the occurrence of the cement hydration reaction, the water in the reservoir is released to further promote cement hydration, and more hydration products are obtained.
4. It could be observed that the total heat released from self-curing UHPC is higher than that from the control group, Z0. In the initial period, the heat release rate of self-curing UHPC increased because of the addition of zeolite sand. As the content of calcined zeolite sand increased, the heat release rate of the mortars accelerated. In the acceleration period, the time at which heat flow peaks of each hydration heat level appeared is in the order of NZ30 > CZ30 > NZ15 > CZ15 > Z0. During the deceleration period, it could be observed that the slope of the heat release rate cures of CZ15 is the same as that of CZ30 with respect to the hydration reaction. The slope of the heat release rate cures of NZ15 is the same as that of NZ30 with respect to the hydration reaction.
5. At the age of 3 days, UHPC has a negative effect due to the addition of zeolite sands with pores, which reduces the compressive strength. However, at the age of 28 days, UHPC with calcined zeolite sand showed a good compressive strength, compared with the control group. This is due to the fact that calcined zeolite sand absorbs more internal curing water, and the internal curing water is released to participate in binder hydration and contributes to the development of compressive strength.

In summary, adding 30 wt.% of calcined zeolite sand as an internal curing agent has a positive effect in reducing the autogenic shrinkage strain of UHPC, without sacrificing compressive strength.

## Figures and Tables

**Table 1:** Chemical compositions of cement, silica fume, and zeolite sand

Materials	SiO_2_	Al_2_O_3_	Fe_2_O_3_	CaO	MgO	SO_3_	ZnO	K_2_O	P_2_O_5_	Loss
Cement (%)	21.65	5.57	2.45	62.68	2.60	2.34	0.11	1.08	0.10	0.46
Silica fume (%)	93.80	0.93	0.56	0.518	0.66	0.23	0.16	1.76	0.08	0.63
Zeolite sand (%)	65.35	13.58	1.61	1.95	1.35					15.8

**Table 2:** Mix proportions of ultra-high-performance concretes (UHPCs)

Number	Binder / %		Sand/ Binder	Water/ Binder	Zeolite Sand / Sand %	Superplasticizer/ Binder %
	Cement	Silica Fume				
Z0	85	15	1.5	0.2	0	3
NZ15	85	15	1.5	0.2	15	3
NZ30	85	15	1.5	0.2	30	3
CZ15	85	15	1.5	0.2	15	3
CZ30	85	15	1.5	0.2	30	3
